# Oxytocin receptor gene expression in the basal forebrain in autism: association with receptor binding levels and single nucleotide polymorphisms

**DOI:** 10.21203/rs.3.rs-7341314/v1

**Published:** 2025-10-05

**Authors:** Ethan E. Dayley, Susan Durham, Michelle C. Palumbo, Jill F. Lundell, Sara M. Freeman

**Affiliations:** Utah State University; Utah State University; Oregon Health & Science University; Massachusetts Institute of Technology; Utah State University

**Keywords:** autism, oxytocin, cholinergic neurons, basal forebrain, nucleus basalis, ventral pallidum

## Abstract

**Background:**

The brain’s oxytocin system has been implicated in the neurobiology of autism (ASD), given the role of oxytocin in modulating social function in humans and animals more broadly. Previous work from members of our group reported dysregulation in oxytocin receptor (OXTR) binding in postmortem tissue from the basal forebrain in donors with autism compared to unaffected control donors. This study follows up on those findings by investigating the potential genetic and gene expression changes that could be driving those differences.

**Methods:**

We used adjacent sections from the same specimens from our previous study and performed duplex fluorescent *in situ* hybridization to visualize and quantify *OXTR* mRNA in the ventral pallidum (VP) and in the cholinergic magnocellular neurons of the nucleus basalis of Meynert (NBM), visualized with choline acetyltransferase (*ChAT*). We genotyped the brain samples using a SNP microarray on extracted DNA. We then used regression models to test associations between OXTR binding density, *OXTR* mRNA levels, and relevant *OXTR* SNPs. Additionally, we tested for correlation between age and *OXTR* mRNA.

**Results:**

ASD specimens showed significantly greater *OXTR* mRNA than unaffected donors in both the VP and the NBM. Furthermore, this is the first demonstration of *OXTR*expression in the cholinergic neurons of the human basal forebrain; 73% of *OXTR* signal in the images of the *ChAT+* neurons were colocalized with the cholinergic neurons. OXTR binding levels from our previous study were positively associated with *OXTR* mRNA in the NBM of control specimens but not in ASD specimens, implying potential dysregulation at the level of protein translation or mRNA trafficking in the NBM in ASD. OXTR binding levels were not associated with *OXTR*mRNA in the VP of either group. We genotyped all specimens for three common SNPs in the *OXTR* gene that have been associated with ASD in the literature, but none significantly predicted levels of OXTR binding or gene expression in the NBM or VP.

**Conclusions:**

Taken together, our results contribute to a more nuanced picture triangulating variation in *OXTR* gene sequence, gene expression, protein levels, and human behavior.

**Trial Registration:**

Clinical trial number: not applicable.

## Background

Autism spectrum disorder (ASD) is a pervasive neurodevelopmental disability that is characterized by persistent differences in communication, sensory sensitivity, and rigid adherence to routine (1). ASD prevalence estimates haven risen significantly over the past few decades, with current prevalence for children in the US estimated at about 1 in 36 (2). This rise in estimated prevalence is controversial, with some studies suggesting that shifts in environmental factors might be driving a true shift in prevalence, while others suggest that new institutional changes in diagnostic criteria are simply driving an increase in awareness (3). Regardless of the reason, the rise highlights the necessity of basic research into the etiology of ASD. Mental health struggles are unfortunately common in the autistic community and may contribute to low rates of both employment and independent living for autistic individuals (4,5). By developing our understanding of the biological processes that create differential outcomes and behaviors for autistic individuals, we may be better able to improve life outcomes for many individuals with autism.

The genetics of autism have been extensively studied, with multiple large-scale research projects giving insight into heritability and genetic associations. Autism is known to have a strong genetic component, with heritability estimates from large-scale studies ranging from 50–90% (6,7). Autism is also known to be polygenic and most likely involves interactions from both rare and common variants (8,9). Meta-analyses of autism genetics have revealed several significant risk alleles, including some associated with the oxytocin receptor gene (*OXTR*) (8,10). Because the oxytocin (OXT) system is known for its role in social interaction (11), which is one of the core symptoms of ASD, studying the oxytocin receptors (OXTR) of the brain is especially relevant to improving our understanding of the neurobiology of ASD.

Given its association with ASD, the OXT system has become a target for treatment of the social symptoms of ASD in many studies over the past two decades. Intranasal oxytocin (IN-OXT) administration has been a particularly active area of study, albeit with somewhat mixed results. Recent meta-analyses suggest that IN-OXT has beneficial effects on social functioning without apparent adverse effects (12,13). That said, the effects appear to be specific to social symptomology of ASD, with little to no effects on non-social domains. It should be noted that camouflaging or “masking” behaviors may complicate interpretation of behavioral studies since external behaviors might not correlate with internal state (14,15). However, fMRI studies have consistently shown differential brain network activation in autistic individuals following IN-OXT administration (16). Affected regions included the amygdala, basal ganglia, frontal and prefrontal cortex, cingulate cortex, and parts of the occipital, temporal and parietal lobes (16). Although it is still unclear to what degree IN-OXT administration affects OXT levels in the cerebrospinal fluid (17,18), the studies above provide strong evidence for the involvement of the OXT system in ASD.

The strong evidence of a link between ASD and OXT highlights the importance of direct brain studies to contextualize IN-OXT studies and better understand what neural circuits may be involved. A 2018 study from members of our group examined OXTR binding density in the basal forebrain and midbrain and found significant differences between autistic and allistic (AST; non-clinical, unaffected) individuals within two brain regions: the nucleus basalis of Meynert (NBM) and ventral pallidum (VP), which are regions associated with visual attention and the mesolimbic reward pathway, respectively (19). In the NBM, OXTR density was significantly increased in autistic donors, whereas in the VP, OXTR density was significantly decreased in ASD compared to AST. These results suggest that autistic individuals are differentially sensitive to OXT within those regions, which could have behavioral implications, although it is speculative at this point. For instance, since the VP is part of the mesolimbic reward pathway, a reduced ability of OXT to activate OXTR in that area may contribute to a reduced experience of social reward or social motivation in autism. The NBM is a cholinergic region of the basal forebrain that has been implicated in selective and sustained visual attention and has projections to multiple other brain regions, including the amygdala and cortex (20), both of which show altered activation following IN-OXT administration (16). Increased OXTR density (and thus sensitivity to OXT) of the NBM could contribute to the differences in social attention that have been reported in ASD (21–23).

To follow up on these results, the current study seeks to examine whether *OXTR* gene expression shows the same pattern of differential levels in ASD compared to AST as its protein product. We used adjacent sections from the same postmortem specimens as the 2018 study to visualize and quantify *OXTR* mRNA transcripts using fluorescence *in situ* hybridization. This approach is unique because most studies of *OXTR* expression in the human brain have relied on a single open-source transcriptomic data set from six allistic donors (24,25), and to our knowledge, the studies to date quantifying *OXTR* expression in postmortem brain tissues from donors with ASD have used qPCR on tissue homogenates (26,27). Both of these approaches sacrifice cellular neuroanatomy, which we have preserved in the current study by working with tissue sections. We hypothesized that *OXTR* mRNA expression in the NBM and VP would align with our receptor binding results and show the same group differences that we previously described between ASD and AST. We also analyzed DNA from all specimens for three *OXTR* single nucleotide polymorphisms (SNPs) implicated in risk for ASD (rs2268491, rs2268495, rs237885) in an effort to link genetic variation with gene expression and protein density.

## Materials and Methods

### Specimens and tissue preparation

A total of 44 frozen blocks of de-identified, unfixed, frozen postmortem human brain tissue from the basal forebrain containing the NBM and/or VP were previously provided by the University of Maryland Brain and Tissue Bank, a brain and tissue repository of the NIH NeuroBioBank. Out of these samples we selected a total of 17 ASD and 24 AST specimens (41 total) for analysis based on presence of identifiable NBM or VP regions in the 2018 study. The specimens were stored at −80°C and had previously been brought to −20°C for cryo-sectioning at 20 μm. Sections were mounted to Fisher Superfrost-Plus slides, sealed in a slide box with a desiccant packet, and returned to −80°C storage until use in fluorescent *in situ* hybridization.

### Fluorescent in-situ hybridization (fISH) assay

To visualize *OXTR* mRNA within the NBM and VP we used the RNAScope^®^ Multiplex Fluorescent v2 Assay (28) according to the manufacturer’s instructions (Advanced Cell Diagnostics, Inc) for unfixed, frozen brain tissue, with some modifications. Sealed slide boxes were thawed for 1 hour at room temperature (RT) before opening. Sections were fixed overnight in 4% paraformaldehyde (pH 7.4) at 4°C. After washes and dehydration in ethanol, endogenous peroxidases were quenched with 0.3% H_2_O_2_ for 10 min at RT. Slides were then boiled for 10 min in the Target Retrieval Reagent, rinsed in ddH_2_O, then incubated for 30 min at 40°C in RNAScope Protease Plus before probe hybridization. We used pooled multiplex probes containing a probe for the human *OXTR* gene and a probe for the human choline acetyltransferase gene (*ChAT*), to colocalize *OXTR* in the cholinergic neurons of the basal forebrain. This approach allowed us to specifically quantify *OXTR* expression in the cholinergic neurons of the NBM as well as in the surrounding basal forebrain and VP. Positive control probes (targeting housekeeping genes *PPIB* and *Polr2a* genes) and negative control probes (targeting bacterial gene *dapB*) were used on adjacent sections from every specimen. Probes were amplified according to manufacturer’s instructions then visualized with distinct Opal dyes that do not overlap in their emission spectra: Opal 570 and 690 (equivalent to Cy3 and Cy5.5, respectively). *OXTR* was visualized with Opal 570 (red) and *ChAT* was visualized with Opal 690 (far-red, or pink). Through optimization experiments using postmortem human brain tissues, we have learned to avoid the use of any green fluorescent dyes (GFP/Opal 570) due to autofluorescence in this spectral range from fixatives or lipofuscin, which accumulates in aged human tissues (29). Prior to coverslipping, cell nuclei were stained with DAPI, a standard fluorescent marker of cell nuclei. All slides were kept in the dark at 4°C after coverslipping to minimize fluorescent signal loss before quantitative imaging. During the assay, two samples had their pooled experimental probes accidentally mixed with negative or positive control probes and had to be excluded from the study. Final sample sizes for our two groups were: 17 ASD and 22 AST.

### Imaging

All slides were stored in the dark at 4°C for an average of 30 days (range of 20–52) prior to imaging. A fluorescence microscope (BZ-X810, Keyence, Itaska IL, USA) was used to image all slides. For all images, we set magnification at 20x with 40% excitation light and standard resolution. Exposure times were set to 1/5 s for DAPI, 1/4 s for *OXTR*, and 3 s for *ChAT*. Because the red and far-red coloration scheme of our selected Opal dyes were difficult to distinguish visually, we chose to change the microscope’s pseudocoloration settings for each channel so that *OXTR* mRNA appears green and *ChAT* mRNA appears red. For quantification, our target number of images per specimen was four per region of interest, and we captured two to five images each from the NBM and VP for each specimen, depending on the size of the region. We also captured images showing the neuroanatomical location of each quantifiable image based on the DAPI, *ChAT*, and *OXTR* signals for later quality control and traceability. We additionally captured a larger image of each sample at 4x magnification for most specimens and compared these to the acetylcholinesterase counterstained images and OXTR film autoradiograms from the original study to confirm our selected neuroanatomical locations for mRNA quantification and ensure that they were taken in the corresponding region to where OXTR binding had been quantified for NBM and VP in our 2018 study.

### Quantification

Images were checked for quality and were excluded for one of two reasons. The first reason was non-matching histology. For the NBM, image inclusion required the presence of cholinergic magnocellular neurons (identifiable by the presence of condensed *ChAT* signal surrounding cell nuclei). For the VP, image inclusion required being located below the anterior commissure according to brain atlas images, appearing as homogeneous tissue without white matter tracts or other significant intrusions, and the presence of *OXTR* mRNA and absence of significant *ChAT* mRNA signal. Two samples for the NBM and two for the VP were dropped from the analysis due to aberrant or unidentifiable histology. The second potential reason for exclusion was incorrect imaging parameters. These were verified both by visual inspection and by checking image metadata. One VP sample was excluded due to incorrect imaging parameters. Two more samples were also accidentally lost. Our final sample sizes were 30 NBM samples (12 ASD and 18 AST), and 21 VP samples (7 ASD and 14 AST). Of these, 26 NBM (11 ASD and 15 AST) and 18 VP (6 ASD and 12 AST) samples had corresponding autoradiography data from the previous study and comprised our final full dataset.

Because our study was conducted across three assays (due to limitations in the number of slides that can be processed together in a single assay), we also checked for batch-wise differences across assays that could have influenced our results. We used an ANOVA to compare brightness for each channel across assays and found no significant differences (Supplemental Materials).

**Figure 1:**
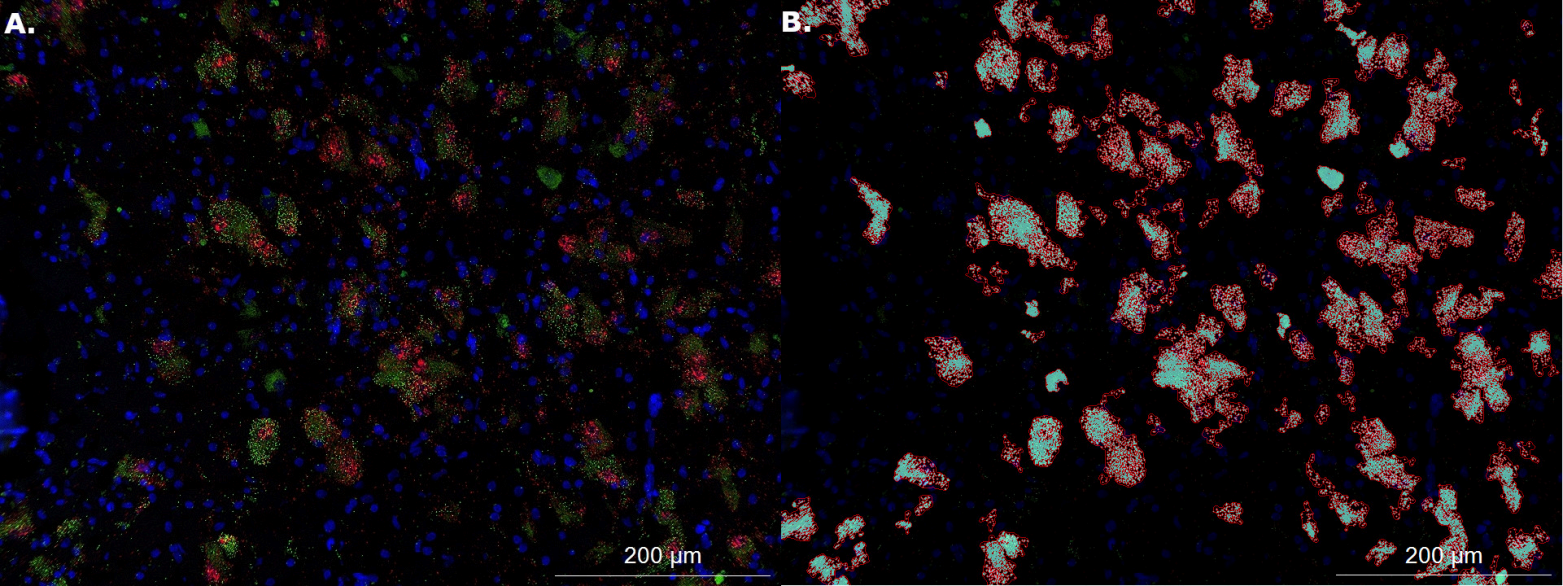
Thresholding image extraction process for quantification. Panel A shows a 20X image with *OXTR* mRNA signal in green and *ChAT* mRNA signal in red. Panel B shows the results of the thresholding process to isolate only *OXTR*signal within the boundaries of the *ChAT+* areas.

The Hybrid Cell Count function in Keyence BZ-Analyzer software was used to quantify signal brightness from all images. This software uses a channel-by-channel thresholding algorithm to extract fluorescence signal intensity (brightness). We selected signal intensity over pixel area as a metric since differing signal intensity in each pixel corresponds to the amount of mRNA present at that location. Each thresholded mask was made by manually adjusting the sensitivity and tolerance of the mask parameters on one representative image until the pattern of quantified pixel area aligns with true pattern of that channel’s signal on the image being quantified. We then saved those settings as a macro and applied it to all images in order to standardize the quantification approach. All of the resulting masks generated by the saved macros were visually checked to ensure that the quantified pixel area aligned with the visual distribution of signal for every channel for every specimen. For the VP, we extracted mean signal brightness from all 20x images for the *OXTR* channel; we refer to this metric as VP *OXTR*. Since the NBM is defined by its composition of large clusters of cholinergic magnocellular neurons, we used them as a marker for where to image. However, the original study quantified macroscopic differences within the NBM as a whole, because autoradiography is not a technique that provides cellular resolution. So we took three measures of NBM *OXTR* mRNA signal to ensure we didn’t miss potential sources of variation. First, we quantified the *OXTR* signal in the NBM in the same way we quantified it in the VP: across the entire 20x image without taking into account the locations of cholinergic neurons; we refer to this metric as NBM *OXTR*. We also calculated the colocalization of *OXTR* expression with regions of condensed cholinergic signal. To do so, we used the software’s single extraction function to designate a target area for *OXTR* extraction using the *ChAT* channel signal. We then filled cracks to make the cholinergic regions continuous. Next, we extracted the *OXTR* channel brightness from within those cholinergic areas. Once we extracted the *OXTR* brightness values, we divided each by the area of cholinergic signal for that image to adjust for differences in the number and size of cholinergic neurons across images. For a representative example of the *OXTR* extraction process within condensed cholinergic regions see Fig. 1. We named this metric *ChAT + OXTR*. We also took an inverse metric which only examined *OXTR* mRNA signal outside condensed cholinergic regions and named it NBM *ChAT- OXTR*. Percent colocalization to *ChAT* of the total *OXTR* signal was calculated as the ratio of *ChAT + OXTR* to total *OXTR* brightness in the NBM, summing values from each image on a sample-wise basis prior to calculating mean and standard error (across all samples and stratified by neurotype).

### OXTR SNP analysis

We extracted DNA from 42 of our original 44 brain samples using a Qiagen kit (2 specimens were dropped due to tissue integrity issues resulting in unrecognizable anatomy), and the purity and concentration of all resulting DNA samples were confirmed using a fragment analyzer. The concentrations of samples were normalized to 50 ng/μL, and 10 μL of each sample was sent to the Genome Core at the University of California Davis for SNP analysis. We used two, 24-sample Illumina Infinium Global Screening Array (GSA) v3.0 BeadChips. The GSA included over 630,000 known SNPs across the human genome. Of the total SNPs genotyped in our samples, only three in the *OXTR* gene had previously been reported to be associated with an increased risk of ASD: rs2268491, rs2266495, and rs237885 (10). A formal evaluation of our full genome-wide SNP dataset is ongoing; the current study focused only on those three *OXTR* SNPs to assess whether genotype for these loci was associated with *OXTR* gene expression levels or OXTR binding density in our samples.

### Statistical analysis

We regressed each *OXTR* mRNA metric on neurotype (ASD or AST). We checked for normality and homoskedasticity of residuals using the ‘simulateResiduals’ function from the *DHARMa* 0.4.6 R package (30) and found that all four metrics except *ChAT + OXTR* had approximately log-normal distributions, so we log-transformed the three log-normal metrics and refit those models. We then calculated p-values for our linear models and calculated estimated marginal means, standard errors, and 95% confidence intervals based on our models using the *emmeans* R package (31). To evaluate whether OXTR protein levels were correlated with *OXTR* gene expression, we calculated correlation coefficients between OXTR binding density from the original study and *OXTR* mRNA metrics.

To analyze whether any of our three target SNPs were significantly associated with OXTR binding density across our samples, we first checked for linkage disequilibrium between our SNPs using the LDMatrix tool to obtain D’ values for each SNP pairing (32). Since our D’ values were medium to high [0.328–0.625], we opted for ridge (L2-penalized) regression to deal with the multicollinearity issues which linkage disequilibrium can cause. To check for effects by neurotype, we generated separate models for ASD and AST neurotypes, as well as combined models, for both the NBM and VP. For each model, we used *cv.glmnet* to select λ (a tuning parameter which determines the amount of shrinkage) with five-fold cross-validation with the λ which minimized the cross-validation prediction error rate to create an L2-penalized model using *glmnet* (33,34). We used the fitted model to generate R^2^ values and obtained p-values for each beta estimate using permutation tests with 1000 permutations. We corrected the p-values for multiple comparisons using the FDR method (35).

To follow up on age-related findings in our prior study, we tested for correlations between age and *OXTR* mRNA levels using Pearson’s correlations, which were calculated in two ways in both regions of interest: across all subjects and in separate groups by diagnosis.

## Results

### Anatomical characterization of OXTR gene expression in the human basal forebrain

**Figure 2:**
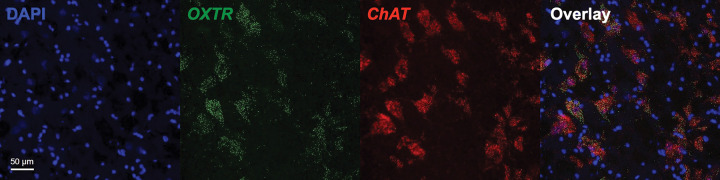
Colocalization of *OXTR* mRNA with the cholinergic neurons of the human basal forebrain. Representative 20X images from across all three imaging channels, plus overlay, showing the high degree of overlap in *OXTR* and *ChAT* gene expression. Scale bar = 50 um.

**Figure 3:**
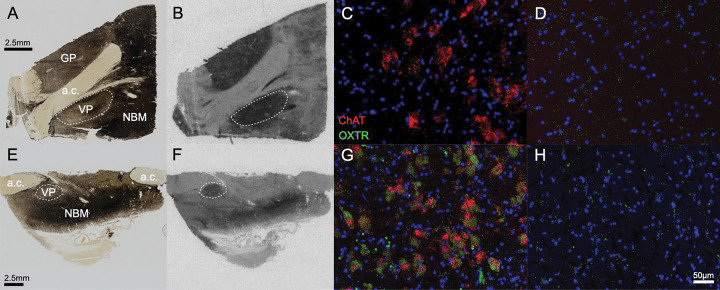
Representative basal forebrain images. Example allistic specimen (A-D) and autistic specimen (E-H). A, E. Acetylcholinesterase stained section, which delineates the heavily cholinergic nucleus basalis of Meynert (NBM) and adjacent ventral pallidum (VP), globus pallidus (GP), and anterior commissure (a.c.). B, F. Film autoradiograms for the sections in A,E, processed for OXTR binding; scale bars align for A-B and E-F. C, G. Representative 20X images of the duplex fluorescence *in situ* hybridization results from the NBM, and columns D and H show the same for the VP; scale bar in panel H applies to all of the fluorescence images, which were all equally processed for *ChAT*(red) and *OXTR* (green) *in situ* hybridization.

We report the first anatomical colocalization of *OXTR* mRNA in the *ChAT* + cholinergic magnocellular neurons of the human basal forebrain (Fig. 2–3). While we also found widespread *OXTR* mRNA throughout the VP, which has interesting implications for future studies of the dopaminergic neurons in this area, our most notable results in the anatomical characterization of *OXTR* gene expression in the basal forebrain are the striking colocalization patterns between *OXTR* and *ChAT +* mRNA. Our use of a *ChAT* probe for duplex fluorescent ISH with *OXTR* was primarily intended to anatomical identify the cholinergic neurons that comprise the NBM, but it quickly became apparent that the high degree of overlap between these two gene expression patterns was a primary outcome of our study. We found that 73 ± 3.8% of the *OXTR* signal in the NBM images was colocalized with the *ChAT* signal. Stratified by neurotype, the percent colocalization for *ChAT +* and *OXTR +* areas was 74.2 ± 5.7% for ASD, and 72.2 ± 5.3% for AST, which are not statistically different.

### OXTR mRNA Comparison Between Neurotypes

**Figure 4:**
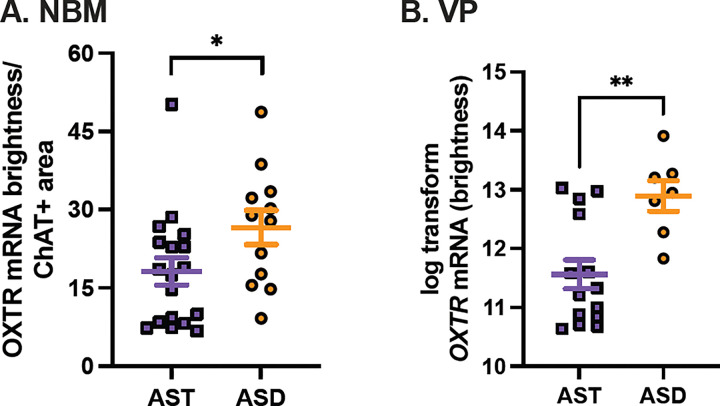
*OXTR* gene expression in the human basal forebrain across groups. A. *OXTR* mRNA signal brightness within the cholinergic areas of the nucleus basalis of Meynert (NBM) was significantly greater in specimens from donors with autism (ASD, orange circles) than allistic donors (AST, purple squares). B. *OXTR* mRNA signal brightness (log transformed for normality) in the ventral pallidum (VP) was significantly greater in ASD specimens than allistic controls. Error bars represent ± SEM. * p<0.05, ** p<0.01.

Only two *OXTR* mRNA metrics had significant differences between neurotypes: *ChAT + OXTR* (p = 0.052) and log-transformed VP *OXTR* (p = 0.003). For both *ChAT + OXTR* and VP *OXTR*, our estimated marginal mean values were significantly higher for ASD than AST (Table 1; Fig. 4).

### OXTR mRNA Association with OXTR Autoradiography

**Figure 5:**
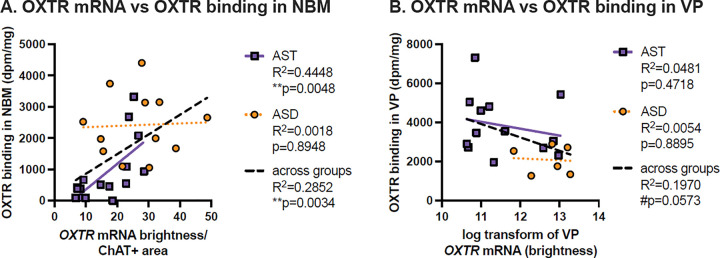
Correlations between OXTR mRNA and OXTR binding across specimens. A. There is a significant positive association across all specimens between OXTR gene expression and receptor binding levels in the nucleus basalis of Meynert (NBM), which appears to be driven by a significant positive association in the allistic (AST) samples only (purple squares). Autistic (ASD) specimens (orange circles) show no correlation between OXTR mRNA and binding. B. There were no significant correlations between OXTR gene expression and receptor binding levels in the ventral pallidum (VP) in either AST or ASD specimens, although there was a trending negative association across all specimens.

In the NBM, there was a significant positive association between *ChAT + OXTR* and OXTR binding across all specimens (slope = 62.81, R^2^ = 0.285, p = 0.003). *ChAT + OXTR* was much more positively associated with OXTR binding for AST (slope = 81.91, R^2^ = 0.445, p = 0.005) than *ChAT + OXTR* in ASD, which was not significantly associated with OXTR binding (slope = 3.964, R^2^ = 0.0018, p = 0.895) (Fig. 5). There were no significant associations between VP *OXTR* and OXTR binding density, although there was a trend toward an overall negative association between VP *OXTR* and OXTR binding density across all samples (slope=−686.3, R^2^ = 0.1970, p = 0.057).

### OXTR Binding Density Association with OXTR SNPs

**Figure 6:**
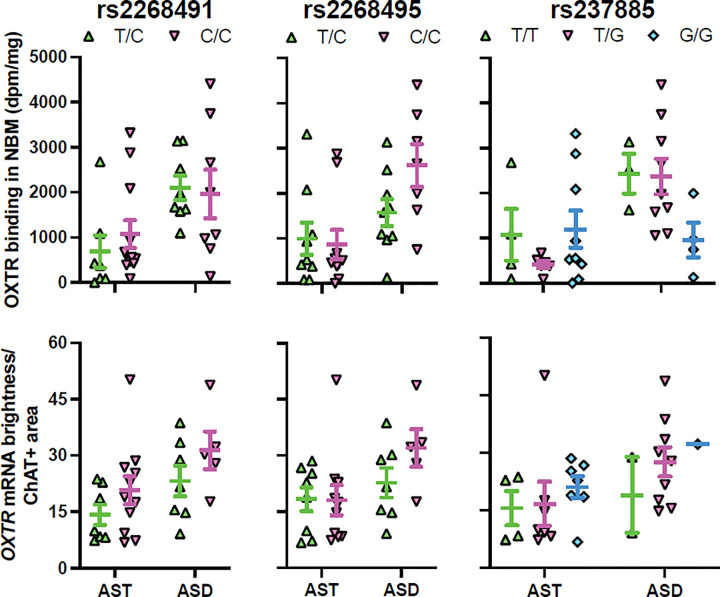
Lack of relationship between genotype and OXTR measures in the nucleus basalis of Meynert (NBM). Top row of graphs shows OXTR binding levels by genotype in the NBM for the three SNPs of interest. The ASD samples initially showed a trend toward a significant effect of genotype for SNP rs2268495, but the result did not survive correction for multiple comparisons. The ASD samples also initially showed a significant effect of genotype for SNP rs237885, which also did not survive correction. Bottom row of graphs show *OXTR* mRNA levels within *ChAT*+ areas by genotype for the three SNPs of interest. There were no significant associations between genotype and *ChAT+ OXTR* mRNA for any of the SNPs. Error bars represent ± SEM.

**Figure 7:**
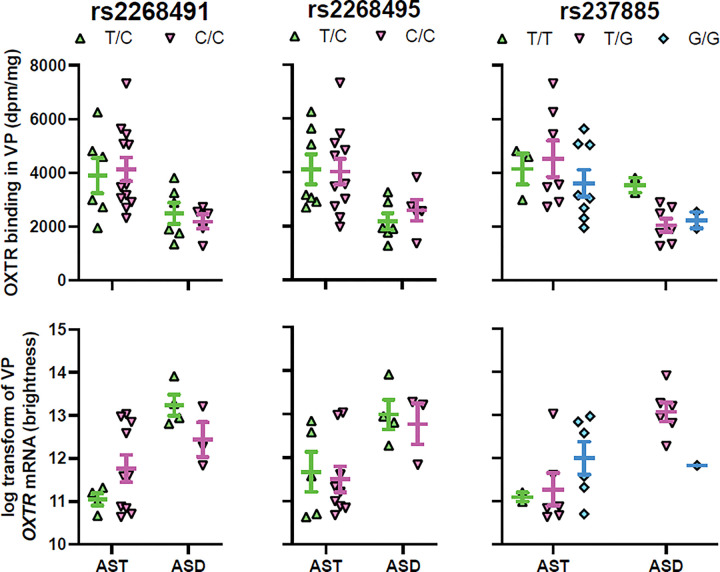
Lack of relationship between genotype and OXTR measures in the ventral pallidum (VP). Top row of graphs show genotype by OXTR binding levels in the VP for the three SNPs of interest. The ASD samples had a trend toward an effect of genotype on OXTR binding levels for rs237885, which did not survive statistical correction. There were no significant associations between genotype and *OXTR* mRNA in the VP for any of the SNPs. Error bars represent ± SEM.

Two of our models yielded associations worth noting. For the NBM OXTR (Fig. 6) with only ASD samples (R^2^ = 0.33), rs2268495 had a p-value of 0.057 (adjusted p-value = 0.228), and rs237885 had a p-value of 0.028 (adjusted p-value = 0.224). For the VP OXTR (Fig. 7) with ASD only (R^2^ = 0.90), rs237885 had a p-value of 0.092 (adjusted p-value = 0.658). But none of the correlations survived correction for multiple comparisons, as seen in the adjusted p-values reported above.

### OXTR mRNA Association with OXTR SNPs

No significant correlations between our SNPs of interest and *OXTR* mRNA were found in either the NBM (Fig. 6) or VP (Fig. 7).

### OXTR mRNA Correlation with Age

**Figure 8:**
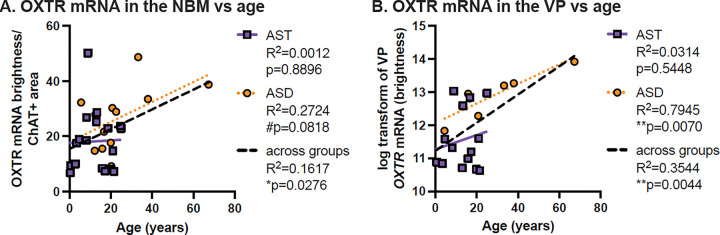
OXTR gene expression increases with specimen age, especially in autism. In both of our regions of interest, we found a significant positive association between OXTR mRNA levels and donor age. In both regions, this appears to be driven by ASD specimens, which showed a significant positive association in the VP, but only a trending association in NBM.

*OXTR* mRNA was significantly positively correlated with age for both the NBM and VP (Fig. 8). Our Pearson’s correlation for *ChAT + OXTR* mRNA with age was r = 0.402 with significance p = 0.028, and for VP *OXTR* mRNA was r = 0.813 with significance p = 0.004. Correlation by diagnosis in the NBM (Fig. 8A) resulted in a trending correlation for *ChAT + OXTR* mRNA with age (r = 0.522; p = 0.082) for ASD only, and no significant correlation for AST (r = 0.035; p = 0.900). For the VP (Fig. 8B), correlation by diagnosis revealed a significant association for ASD (r = 0.961; p < 0.01), and no significant correlation for AST (r = 0.214; p = 0.463).

## Discussion

This study sought to evaluate whether there are differences in *OXTR* gene expression or genotype between ASD and AST samples in two regions of interest in the human basal forebrain that were previously reported to have differential levels of OXTR protein, as measured by receptor binding density. We found increased *OXTR* mRNA in both the NBM and VP of ASD specimens. The NBM result aligns with our previous finding of higher OXTR binding in the NBM in ASD, but the VP result is counter to our initial hypothesis that the gene expression levels would match what we previously reported: ASD specimens had lower OXTR binding density in the VP. We had previously interpreted the lower levels of OXTR binding in the VP in the context of reduced social reward or reduced social motivation, given the VP’s role in the mesolimbic dopaminergic reward pathway. But in the current study, we found increased *OXTR* mRNA in the VP in ASD, as well as a lack of association between *OXTR* mRNA and receptor binding in the VP. Taken together this result could indicate dysregulation in the translation of *OXTR* mRNA into functional, mature cell-surface protein in the VP in ASD; however, we also found a lack of association between OXTR mRNA and binding in the VP of AST controls. Although this result was somewhat unexpected, a lack of association between *OXTR* mRNA and binding has recently been reported in the prairie vole brain using the same techniques as the current study (36). While gene expression and protein levels would be expected to co-vary, this discrepancy can be explained by the ability of mRNA in neurons to be transported to axons (37,38), which has been observed in *OXTR* mRNA in rodent hypothalamus(39). In fact, these differences in relative levels of *OXTR* mRNA vs receptor binding across multiple studies and species highlights the importance of using both receptor binding and measurements of *OXTR* gene expression in order to draw strong inferences about the function of OXT in the brain. Future studies are clearly needed to address cellular and molecular characteristics of the OXT/OXTR signaling pathway in a region-by-region approach to assess whether differences in gene expression, protein translation, or receptor internalization are contributing to ASD neuropathology and symptom expression.

Interestingly, *OXTR* mRNA signal brightness within the *ChAT* + areas was the only gene expression metric from the NBM that showed significant differences by neurotype. The fact that the *OXTR* mRNA levels outside of the *ChAT +* areas were not predictive of differences between groups points toward functional consequences of increased *OXTR* gene expression specifically within the cholinergic neurons of the NBM in ASD, rather across the basal forebrain more broadly. This result of increased *ChAT* + *OXTR* mRNA levels aligns with our findings and interpretation from our previous study: it is possible that increased *OXTR* gene expression and increased OXTR binding in this area of the brain in ASD underlies an increased sensitivity of the cholinergic basal forebrain to OXT release, which could in turn be related to difficulties with social visual attention, because the basal forebrain’s cholinergic input to the neocortex mediates selective and sustained attention to visual stimuli (40). However, when we investigated whether *OXTR* mRNA levels in *ChAT +* areas were correlated with OXTR binding densities measured in anatomically adjacent sections from the same specimens, there was no significant association between these OXTR measures in the ASD specimens, while there was a significant positive association in AST. Thus, it appears that as *OXTR* gene expression increases in the NBM in unaffected controls, so does the mature cell surface OXTR receptor protein, but not in ASD samples. We interpret this collection of results to imply overall elevated OXTR production in ASD but possibly some dysregulation in protein translation or possibly in receptor internalization and turnover, resulting in high but disconnected levels of both mRNA and protein. Imbalances in gene expression levels for molecules involved in OXT secretion (CD38) and breakdown (LNPEP) have recently been reported in ASD (41), which sets a precedent for dysregulation in the OXT system, from both the peptide and receptor sides.

There were no effects of genotype at any of the three SNPs on *OXTR* gene expression or OXTR binding levels in either of our two regions of interest. Each of the three SNPs that we investigated (rs2268491, rs2268495, rs237885) are intronic and have been associated with ASD across studies, and two of these (rs2268491, rs237885) emerged as significant in the largest meta-analysis of *OXTR* and ASD to date (10). So our lack of any significant findings here is likely due to sample size. Although our study is the first to our knowledge to attempt to link *OXTR* genotype directly to neural OXTR phenotypes in tissue sections with preserved anatomy, it is likely statistically underpowered to detect differences. Additionally, because the specific SNP BeadArray we used only allowed us to assess *a priori* SNPs, the other known *OXTR* SNPs that have been associated with ASD were not measured.

We found a significant positive correlation between *OXTR* mRNA and age in both the VP and NBM across all specimens. Interestingly, in our previous binding study, we only found a significant association between OXTR levels and age in the VP (not NBM), and the direction of the association was opposite: OXTR binding was highest in early childhood and was negatively associated with increasing age across all specimens. In our current study, it appears that the relationship in the NBM between age and *OXTR* mRNA were being driven entirely by one specimen from a 67 year old donor with ASD; when this data point is removed, the association is no longer significant. However, when that donor is removed from the VP dataset, the significant positive association between OXTR mRNA and age remains, both for the ASD subset (R^2^ = 0.6804; p = 0.0433) and for all specimens together (R^2^ = 0.2205; p = 0.0367). Thus, it appears that while OXTR binding may be reduced in the VP in ASD in early childhood and adolescence, *OXTR* gene expression levels in the same region increase in ASD with increasing age.

Given the postmortem nature of this study, our sample size is lower than would be expected for similar studies of living subjects. However, compared to most histological studies of the postmortem brain of humans and nonhuman primates, our sample size is double to triple what is typical in those studies. Due to unavailability of clinical data associated with our specimens, we were unable to evaluate whether symptom severity was associated with any of our outcome measures.

## Conclusions

The group differences observed in this study provide a more nuanced picture of the ways in which the OXT system interacts with ASD and opens the door to future studies examining post-translational transport, modifications and degradation of OXTR. Beyond the assessment of neurotype group differences, the resulting colocalization of *OXTR* mRNA in the cholinergic neurons in the human basal forebrain is striking. To our knowledge this is the first demonstration of *OXTR* mRNA in the cholinergic neurons of the human basal forebrain, a result that would not have been possible through the use the transcriptomic methods used in brain tissue homogenates. This result provides a critical piece of anatomical information to the “puzzle” of understanding OXT’s function in the human brain. By showing that the cholinergic magnocellular neurons of the human basal forebrain are expressing high levels of OXTR mRNA, our results provide a mechanism by which OXT released in the basal forebrain can directly bind to and modulate the function of the neurons that provide cholinergic input to the neocortex. This critical anatomical piece of evidence supports the idea that OXT is likely acting in the human brain as a “modulator of modulators”—an idea that has been increasing in popularity over the last few years (42–45). As differences in social attention can contribute to more substantial differences in social perception and social cognition, future studies should assess the interaction between oxytocin and the cholinergic system in the modulation of behavior, similar to how studies of oxytocin’s interplay with dopamine (46) and with the opioid system (47,48) are now contributing to a more complex understanding of the mechanisms by which OXT can impact physiology and behavior.

## Supplementary Material

Supplementary Files

This is a list of supplementary files associated with this preprint. Click to download.

• AppendixS1.pdf

## Figures and Tables

**Table 1:** Estimated marginal means and confidence intervals for *OXTR*mRNA metrics

Region	Neurotype	Mean	Standard Error	95% Confidence Interval Lower Bound	95% Confidence Interval Upper Bound
NBM	Autistic	26.6	3.21	20	33.2
	Allistic	18.2	2.62	12.8	23.5
VP	Autistic	398486	127064	204441	776711
	Allistic	105045	23685	65527	168394

Metrics are ChAT + OXTR and VP OXTR for NBM and VP respectively.

## Data Availability

The datasets used and/or analysed during the current study are available from the corresponding author on reasonable request.

## Abbreviations

ASD: Autism Spectrum Disorder
AST: Allistic
ChAT: Choline Acetyltransferase
fISH: Fluorescence In-Situ Hybridization
GSA: Illumina Infinium Global Screening Array
IN-OXT: Intranasal Oxytocin
IRB: Institutional Review Board
NBM: Nucleus Basalis of Meynert
OXT: Oxytocin
OXTR: Oxytocin Receptor
RT: Room Temperature
SNP: Single-Nucleotide Polymorphism
USU: Utah State University
VP: Ventral Pallidum
